# Networking to Improve Nutrition Policy Research

**DOI:** 10.5888/pcd12.150329

**Published:** 2015-09-10

**Authors:** Sonia A. Kim, Heidi M. Blanck, Angie Cradock, Steven Gortmaker

**Affiliations:** Author Affiliations: Sonia A. Kim, Heidi M. Blanck, Division of Nutrition, Physical Activity, and Obesity, Centers for Disease Control and Prevention, Atlanta, Georgia; Angie Cradock, Steven Gortmaker, Harvard T. H. Chan School of Public Health, Boston, Massachusetts.

## Abstract

Effective nutrition and obesity policies that improve the food environments in which Americans live, work, and play can have positive effects on the quality of human diets. The Centers for Disease Control and Prevention’s (CDC’s) Nutrition and Obesity Policy Research and Evaluation Network (NOPREN) conducts transdisciplinary practice-based policy research and evaluation to foster understanding of the effectiveness of nutrition policies. The articles in this special collection bring to light a set of policies that are being used across the United States. They add to the larger picture of policies that can work together over time to improve diet and health.

## Introduction

The dietary quality of many Americans is poor and, combined with low levels of physical activity, contributes to early death and disability from diseases such as obesity, diabetes, cardiovascular disease, and certain cancers (1). To improve diet, the public health community has recognized the need for a range of approaches that span the socioecological model and take into account the interaction between the environment and the individual in making food choices (2–5). A key notion to this interplay is that people’s learned food preferences along with social, information, and food environments are powerful influences on dietary intake (5). Effective nutrition policies may affect these environments in various ways, including enabling people to acquire healthy food preferences or removing barriers to healthy choices (5). For example, early care and education, school, and worksite food standards can repeatedly expose people to healthy food offerings, a factor important for the development of food preferences (6). Although the potential of policy strategies to improve healthy food environments and human diet is recognized, this field is in its nascent stages (7).

In 2009, The Centers for Disease Control and Prevention’s (CDC’s) Division of Nutrition, Physical Activity, and Obesity created the Nutrition and Obesity Policy Research and Evaluation Network (NOPREN; www.nopren.org). NOPREN’s goal is to foster understanding of the effectiveness of policies to improve the physical food environment and the food-related economic, social, and information environments. NOPREN members conduct transdisciplinary practice-based policy research and evaluation using a framework that includes policy identification, development, implementation and outcomes, and translation and dissemination, as previously described (8). Researchers consider a variety of policy levers (eg, legislation, regulation, executive orders, and zoning) at the national, state, territorial, tribal and community levels. 

The initial NOPREN core of 5 funded Prevention Research Centers (PRCs) and their CDC technical advisors has expanded membership to realize the benefits of working as a network, thus leveraging expertise, funding, resources, and relationships. Additional members now include universities not funded by NOPREN, staff from state and local health departments, education and child health agencies, and nonprofit organizations. Key partners are Robert Wood Johnson Foundation’s Healthy Eating Research (HER) Program and the National Collaborative on Childhood Obesity Research (NCCOR). NOPREN’s technical advisors include staff members from CDC and the National Institutes of Health (8).

NOPREN members work collaboratively through multidisciplinary working groups that address priority areas such as access to drinking water, access to food in rural areas, the impact of policy research, food policy councils, school wellness programs, and early child care and education. Working group members share tools, develop topic-specific capacity, and conduct multisite coordinated research and evaluation. These collaborations are reflected in a collection of NOPREN articles, many of which are products of a working group. 

## An Overview

The articles in this special collection span multiple policy levels (national, state, local); types (executive order, guidelines and recommendations, legislation); and settings (urban and rural communities, early child care and education). They address several components of NOPREN’s evaluation framework. Articles by Quinn et al (9) and Walsh et al (10) demonstrate in-depth understanding of policy development and adoption. Careful examination of policy implementation and its effects on the food and beverage environment are found in articles by Cradock et al (11) and Ritchie et al (12). 

The transferability of policies from urban settings to rural communities (13) is discussed in the work of Calancie and colleagues. The translation, communication, and dissemination of policy research and best practices (14) are addressed by Otten et al. Making use of diverse methodologies (eg, systematic review, qualitative case design, quantitative survey analysis), these studies provide a broader understanding of the potential role of policy as a strategy to support healthier diets.

## Policy Development and Adoption

Researchers at the University of Washington used a qualitative case study design to examine the development and reach of an innovative policy approach to healthy food access adopted by a local board of health (9). The King County Local Board of Health (in Washington State) developed guidelines for healthy vending, using a newly adopted policy mechanism that allowed for greater specificity without the complexity of a regulation (9). Other communities may benefit from understanding the array of policy tools being used and the feasibility and benefits of these tools.

The importance of considering local context emerged as a theme from a case study by Walsh and colleagues of the role of the Cleveland-Cuyahoga County Food Policy Coalition (CCCFPC) in 4 policy efforts to improve Cleveland’s urban food environment (10). Researchers found that the stimulus for the policies originated with citizens, and the CCCFPC was instrumental in getting those citizens’ needs heard. The CCCFPC’s role in educating policy makers and its relationships with diverse partners were key elements in the adoption of these policies (10).

### Policy implementation

Development and adoption of a policy are often early steps toward creating healthy environments but may not guarantee complete adherence to a policy. Therefore, implementation of policies and their resulting effects on the food and beverage environment should be carefully considered as part of policy research and evaluation.

Using a pre/post natural experimental design, Cradock and colleagues examined the impact of Boston’s Healthy Beverages Executive Order (HBEO) on the availability of healthy beverages in Boston City agency locations (11). The HBEO, which took effect in 2011, required Boston agencies to eliminate the sale of sugar-sweetened beverages on city property. Investigators found that 2 years after the HBEO was implemented, the average proportion of sugary beverages available per access point had significantly decreased, and city agencies were more than 4 times as likely to offer only healthier beverages as they were before the HBEO, but not all retail points were in full compliance (11). Similarly, Ritchie et al found that the provision of water to children by California-licensed childcare providers increased after the implementation of federal and state policies addressing the issue (12). However, not all childcare providers were compliant, demonstrating that policy adoption is important but not sufficient to the creation of healthier environments. These studies emphasize the need for monitoring implementation and adherence to policies.

Another aspect of policy evaluation is understanding whether a policy addresses the needs and circumstances of the target population. A 2008 New York City policy established 1,000 permits for mobile fruit-and-vegetable vendors (aka Green Carts) to operate in neighborhoods with the least availability of healthful foods (15). Many residents in these low-income neighborhoods rely on Supplemental Nutrition Assistance Program (SNAP) benefits for food purchases. Beginning in 2010, the New York State Department of Health funded electronic benefit transfer machines so that residents could use their SNAP benefits at the carts. Researchers from New York University found that customers using SNAP benefits at Green Carts spent on average $3.86 more per transaction than those who paid with cash, suggesting that the policy did affect the intended population (15).

## Translation, Communication, and Dissemination

NOPREN’s evaluation framework includes activities related to the translation, communication, and dissemination of policy-relevant research. These activities may include characterizing the potential for transferability of policies; translating and disseminating best practices for policy implementation; and ensuring that research findings are communicated to relevant stakeholders.

Little is known in the US public health community about how evidence from nutrition research is used in policy development and how researchers communicate their findings to policy makers (14). Otten and colleagues addressed this gap through interviews with public health and nutrition researchers, finding a wide range of practices, barriers (mainly in academic settings), and facilitators (including the “desire to make a difference,” collaborations, and mentorship) (14).

Results from an examination of the nutrition-related practices and attitudes of child care providers among licensed family child care homes (FCCH) in Rhode Island by Tovar and colleagues (16) demonstrate the need for increased cultural sensitivity in such settings. The authors suggest that culturally and linguistically relevant trainings that are tailored for FCCHs (rather than to child care centers) are needed to make certain that all children receive adequate nutrition (16). They also point out that training on policies and practices that enable children to learn healthy food preferences and eating behaviors must be expanded if dietary changes are to be equitable and sustainable (16).

As discussed by Calancie et al (13), much of the policy research and evaluation on nutrition and obesity has been done in urban settings. However, rural residents often face disparities in obesity-related health outcomes and risk factors (17–19). NOPREN’s Rural Food Access Working Group examined the implementation and adaptation of nutrition and obesity policies for rural settings (13). This assessment illuminates strategies for overcoming barriers to healthy food availability in rural areas.

## Conclusions

NOPREN conducts research relevant to developing a culture of smart food policy. NOPREN’s expansion of working groups, including a new Hunger Safety Net group in 2015, and strategic partnerships are responses to the need for policy research in emerging areas of importance. NOPREN serves as a forum where members can learn the latest theories and research in the field, share and collaborate on tools and methods, and develop capacity to conduct policy research relevant to practitioners and policy makers and responsive to communities most in need. This collection of articles on policy research brings to light policies that are being tried across the country and adds to our knowledge about which policies can work to improve the US diet.
